# Optimizing the operation strategy of a combined cooling, heating and power system based on energy storage technology

**DOI:** 10.1038/s41598-023-29938-6

**Published:** 2023-02-20

**Authors:** Yu Zhang, Yan Deng, Zimin Zheng, Yao Yao, Yicai Liu

**Affiliations:** School of Energy Science and Engineering, Central South University, Changsha, 410083 China

**Keywords:** Energy science and technology, Engineering

## Abstract

Energy storage technology is the key to achieving a carbon emission policy. The purpose of the paper is to improve the overall performance of the combined cooling, heating and power-ground source heat pump (CCHP-GSHP) system by the battery. A new operation strategy (the two-point operation) is proposed by controlling the power generation unit work. The power generation unit has two operation modes of non-operation and rated efficiency operation by the storage electricity battery. The new operation strategy is compared with the traditional CCHP-GSHP that without a battery. The optimization goals include the primary energy saving ratio, the reduction ratio of carbon dioxide emissions, and the annual total cost saving ratio. The independent GSHP system is used as a reference system. Multipopulation genetic algorithms are selected to achieve the problem of optimization. A hotel building is selected for a case study. The optimal configuration of the coupling system is computed following the electric load strategy. Finally, the results show that the CCHP-GSHP system has a better performance under the new operation strategy compared with the traditional CCHP-GSHP (the primary energy saving ratio increases by 5.5%; the annual carbon dioxide emission reduction ratio increases by 1%; the annual total cost reduction ratio increases by 5.1%). This paper provides reference and suggestions for the integration and operation strategy of CCHP-GSHP in the future.

## Introduction

With the situation of energy and the environment becoming more and more serious, energy saving and emissions reduction have received increasing attention^1^. The CCHP system can achieve multi-stage utilization of energy and effectively decrease the carbon emissions^2^. Its advantages have led to its rapid development in energy savings and environmental protection^3^. Nojavan et al.^4^ implemented renewable energy in a microenergy grid to model this system. Zeng et al.^5–7^ used a hybrid particle swarm optimization algorithm and genetic algorithm to dynamically optimize the CCHP system considering the nonlinearity of the equipment. Considering the equipment capacity and energy allocation of the system, the optimization results were verified by comparison with the traditional system in three aspects: energy saving rate, carbon dioxide reduction ratio, and annual total cost. Soheyli^8^ considered a novel CCHP system that included photovoltaic modules, wind turbines, and solid oxide fuel cells as prime moors. Lu et al.^9^ proposed a seasonal operation strategy of the distributed energy system, which used an analytic hierarchy process to determine the weight and differential evolution particle swarm optimization hybrid algorithm to solve the model. Feng et al.^10^ studied the performance of the system from the perspective of different cooling methods and optimized the CCHP system based on a hybrid chiller. Su et al.^11^ optimized and analysed the key operating parameters of the CCHP-GSHP system based on the comprehensive benefits of economy, energy conservation, and environmental protection. Chu et al.^12^ considered carbon tax as the objective function and compared the advantages and disadvantages of the CCHP-GSHP joint supply system of different types of buildings. Yan et al.^13^ designed a new CCHP microgrid structure with compressed air energy storage, mainly considering energy utilization and energy cascade utilization. Li et al.^14^ compared the CCHP-GSHP coupled system with a heat exchanger with the CCHP-GSHP system without a heat exchanger. Zhang et al.^15^ compared the advantages and disadvantages of four refrigeration methods, namely waste heat driven absorption refrigerator, electric refrigerator, gas absorption refrigerator, and ground source heat pump, in the CCHP system. Arabkoohsar and Sadi improved the hybrid configuration of a power generation system. The system has good comprehensive performance for reducing carbon dioxide emissions^16^; Sadi et al.^17,18^ analyzed the benefits of using solar energy and biomass energy in India; Shoeibi et al.^19^ analyzed and summarized the application of solar energy in energy systems.

Recent studies have made progress in the integration schemes and optimization algorithms of CCHP systems. However, there are still many problems including to modifying the system configuration and operation strategy. The point was not more considered that the existence of the storage battery can improve the efficiency of the power generation unit (PGU) in the CCHP-GSHP system. In this paper, the battery is used to store PGU electricity and the PGU adopts the two-point operation strategy for the CCHP-GSHP system. To meet the load fluctuation of the building and enhance the combined performance of the coupled system, the system is equipped with a battery to enhance the regulation ability of the system. It has a positive impact on the improvement of the efficiency of the whole system. The case of a hotel building in Changsha is researched to achieve the optimal configuration. The multipopulation genetic algorithm (MGA) was used for calculation.

## System composition

### Independent ground source heat pump system

**Figure 1:**
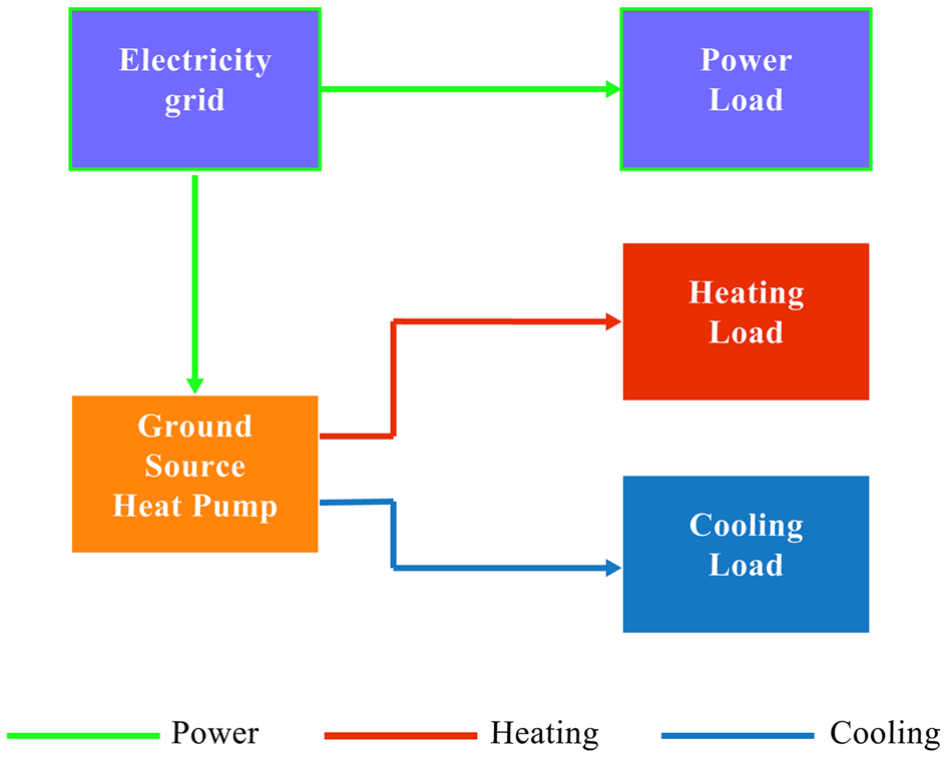
Independent GSHP system.

The GSHP system is considered as a reference system for the design system, and its design results are shown in Fig. 1.

The GSHP system provides the cooling and heating load required by the building. The power required by the building. The power required by the building and the system is provided by the electricity grid. The power supply balance of the system is described as follows:

1 $$E_{grid} = E + E_{GSHP} + E_{GSHP,er} .$$

where $E$ represents the electrical demand of the building, $E_{GSHP,er}$ is the amount of electrical demand to work the whole system, and $E_{GSHP}$ is the electrical load of the driving GSHP system, which can be expressed as:

2 $$E_{GSHP} = Q^{c,h} /COP_{GSHP} .$$

where $Q^{c}$ and $Q^{h}$ denote the cooling capacity and heat capacity of the building, respectively. $COP_{GSHP}$ represents the cooling and heating efficiency of the GSHP system and could be defined as follows^2^:

3 $$COP_{GSHP} = COP_{gshp}^{\max } [a_{2} \eta^{2}_{gshp} + a_{1} \eta_{gshp} ].$$

where $COP_{gshp}^{\max }$ represents the rated efficiency of GSHP, and $\eta_{gshp}$ is the partial load coefficient of GSHP in the reference system, which can be written as:

4 $$\eta_{gshp} = Q^{xy} /Q_{gshp}^{\max } .$$

where $Q_{gshp}^{\max }$ is the rated capacity of the GSHP, the equation represents cooling or heating. Considering energy conversion and transmission, the main energy consumption of the system per hour is

5 $$F_{GSHP}^{{}} = E_{GSHP} /(\eta_{E} \eta_{T} ).$$

where $\eta_{E}$ and $\eta_{T}$ are the generation efficiency and transmission efficiency, respectively.

### Traditional CCHP-GSHP system

**Figure 2:**
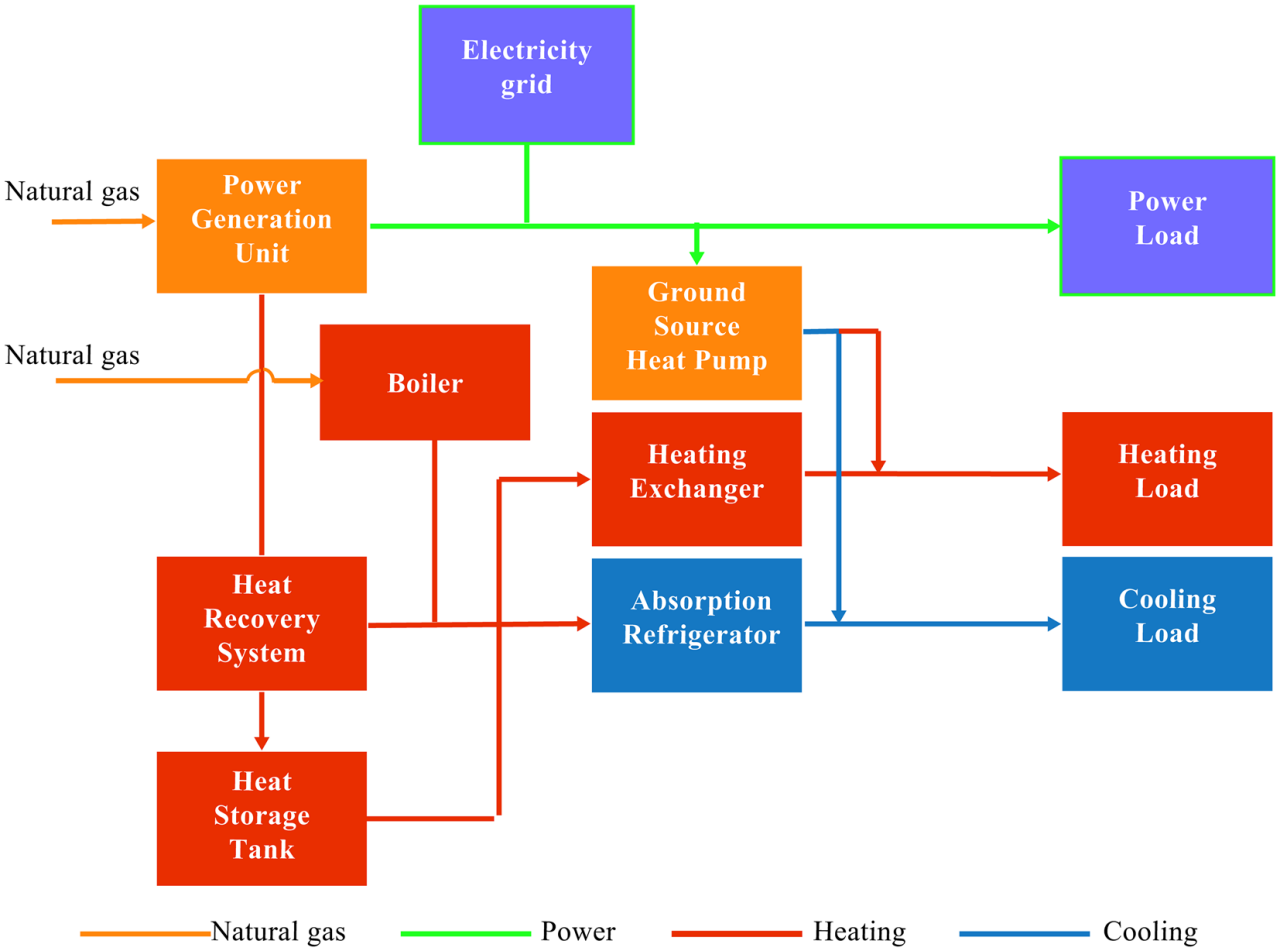
Traditional CCHP-GSHP system.

The traditional GSHP coupled with cooling, heating, and power system is considered as the comparison system of the design system, and its design results are shown in Fig. 2.

The power balance formula of the traditional CCHP-GSHP system is as follows:

6 $$E_{grid} + E_{pgu} = E + E_{er} + E_{gshp}$$

where, $E_{grid}$ is the electricity consumption of the grid, $E_{pgu}$ denotes the power supplied by the PGU, $E$ is the electricity load requirements of the building, $E_{er}$ is the power requirements of the system during operation, and $E_{gshp}$ is the power requirements of the GSHP, which can be defined as belows:

7 $$E_{gshp} = Q_{gshp}^{c/h} /COP_{gshp} .$$

where $Q_{gshp}^{c/h}$ is the cooling or heating supply by the GSHP, and it could be written in Eq. (8):

8 $$Q_{gshp}^{c/h} = mQ^{c/h} .$$

where $m$ denotes the proportion of cooling or heating quantity supplied by GSHP between the cooling or heating load requirements of the system, which determines the flexibility of the system and is chosen as the optimization variable. The ground source heat pump part is the same between the traditional CCHP-GSHP system and the reference system:

9 $$COP_{GSHP} = COP_{gshp}^{\max } [a_{2} \eta^{2}_{gshp} + a_{1} \eta_{gshp} ].$$

where $\eta_{gshp}$ is the partial load coefficient of GSHP, and it could be written in Eq. (10).

10 $$\eta_{gshp} = Q_{gshp}^{c/h} /Q_{gshp}^{\max } .$$

where $Q_{gshp}^{\max }$ is the rated capacity of the GSHP. The rated capacity of the PGU determines whether the system can efficiently realize the cascade utilization of energy. Therefore, it is also chosen as the optimization variable in the system, and the natural gas consumed by the PGU is:

11 $$F_{pgu} = E_{pgu} /\eta_{pgu} .$$

where $E_{pgu}$ is the actual power generation of the PGU and can be expressed as follows^20^:

12 $$E_{pgu} = \left\{ {\begin{array}{*{20}c} 0 & {0 < f < t} \\ {E_{pgu}^{f} } & {t < f < 1} \\ {E_{pgu}^{\max } } & {t < f < 1} \\ \end{array} .} \right.$$

Parameter *t* can effectively improve the power generation efficiency of the PGU and play a decisive role in improving the efficiency of the whole system. Parameter *f* is the partial load coefficient of the PGU and is chosen as the decision variable, which can be written as:

13 $$f = E_{pgu} /E_{pgu}^{\max } .$$

14 $$\eta_{pgu}^{f} = a_{0} + a_{1} f_{pgu} + a_{2} f_{pgu}^{2} .$$

where $E_{pgu}^{\max }$ is the rated power generation of the PGU, and $\eta_{pgu}^{f}$ is the power generation efficiency. The thermal equilibrium of the coupled system can be expressed as:

15 $$Q_{pgu} + Q_{b} + \eta_{tst} Q_{tst}^{out} - \eta_{tst} Q_{tst}^{in} = Q_{ab} /COP_{ab} + Q_{he} /\eta_{he} .$$

16 $$Q_{pgu}^{{}} = F_{pgu} (1 - \eta_{pgu} )\eta_{rec} .$$

17 $$Q_{ab}^{{}} = (1 - m)Q^{c} .$$

where $Q$ stands for the regenerated heat. $\eta_{tst}$ is the heat loss coefficient of the thermal storage tank per hour, and $\eta_{rec}$ is the efficiency of the heat recovery system. $COP_{ab}$ can be expressed as:

18 $$COP_{ab} = d_{3} (\eta_{ab} )^{3} + d_{2} (\eta_{ab} )^{2} + d_{1} (\eta_{ab} ) + d_{0} .$$

19 $$\eta_{ab} = Q_{ab} /Q_{ab}^{\max } .$$

20 $$Q_{he}^{h} = (1 - m)Q^{h} .$$

The gas consumed by the boiler can be calculated as follows:

21 $$F_{b} = Q_{b} /\eta_{b} .$$

### Two-point operation model CCHP-GSHP system with accumulator in prime motor

**Figure 3:**
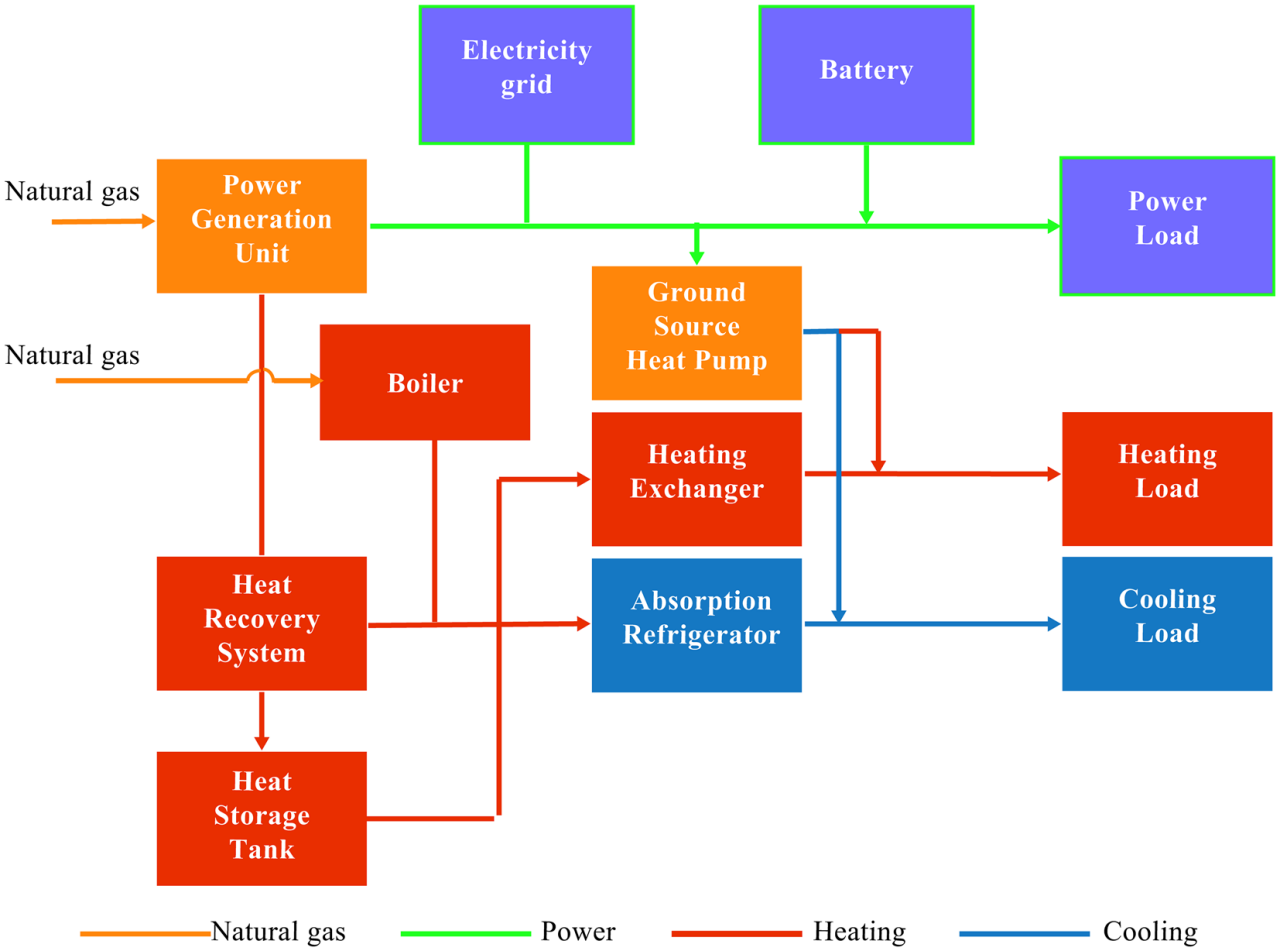
Two-point operation CCHP-GSHP system.

The schematic of the two-operation model CCHP-GSHP system with accumulator in PGU is shown in Fig. 3. The power balance formula of two-operation model CCHP-GSHP system is as follows:

22 $$E_{grid} + \eta_{out} E_{bat}^{out} + E_{pgu} = E + E_{er} + E_{gshp} + \eta_{c} \eta_{in} E_{bat}^{in} .$$

$E_{grid}$ is the electricity consumption quantity of the grid, $E_{pgu}$ is the amount of power quantity that provided by the PGU, and $E$ is the required electricity load of the building. $E_{bat}^{out}$ denotes the exported power quantity from the accumulator, and $E_{bat}^{in}$ is the imported power quantity to the accumulator. $\eta_{in}$ and $\eta_{out}$ are the charging efficiency and discharge efficiency of the battery^21^. In the two-point operation model CCHP-GSHP system, the capacity of the accumulator (*Bat*_max_) determines the regulating ability of the system, and it is adopted as the optimization variable.

A bidirectional inverter is used to connect between the AC and DC buses, with an efficiency $\eta_{c}$^22^. Then the AC from the PGU is converted to DC to charge the battery, and the rest of the equipment model is consistent with the traditional system. The optimization variables include the rated capacity of the PGU and the load rate of the GSHP, while the starting parameters of the PGU are not considered because the operation mode of the PGU has been determined. $E_{pgu}$ is the actual power generation of the PGU, and it can be expressed as follows:

23 $$E_{pgu} = \left\{ {\begin{array}{*{20}c} 0 & {E + E_{er} + E_{gshp} < \eta_{out} E_{bat}^{out} } \\ {E_{pgu}^{\max } } & {E + E_{er} + E_{gshp} > \eta_{out} E_{bat}^{out} } \\ \end{array} .} \right.$$

## Optimization program

### Optimization algorithm

Genetic algorithms, which can also be called standard genetic algorithms, were first proposed by John Holland in 1975. The multipopulation genetic algorithm is based on the standard genetic algorithm. However, it breaks through the framework of only relying on a single population to achieve genetic evolution, and introduces multiple populations to search for the optimal results at the same time. Each population has different control parameters and achieves different search objectives. Different populations influence each other through migrant operators. Manual selection is used to obtain elite individuals. The ultimate best result is the combined effect of coevolution of all populations. Multiple populations, the genetic algorithm, are developed to optimize the rated capacitance of the prime motor and coupling system in the source heat pump heating/cooling load rate and storage capacity for the system decision variables for the overall optimization. The optimization goal considers the energy, environment, economic indicators, using MATLAB software to realize the whole calculation process.

### Optimization objective

1. Primary energy saving rate

The annual primary energy saving rate was selected as the energy index. Coupled system energy consumption includes natural gas consumed by PGU and boilers and fossil fuels consumed by electricity grids. It can be expressed as:

24 $$F_{CCHP} = \sum\limits_{n = 1}^{8760} {F_{pgu}^{n} + F_{b}^{n} } + F_{grid}^{n} .$$

where $F_{CCHP}$ is the annual fossil energy consumption of the coupled system, $F_{pgu}^{n}$ is the natural gas consumption per hour by the PGU, $F_{b}^{n}$ is the amount of natural gas consumption per hour by the boiler, and $F_{grid}^{n}$ is the amount of fossil fuel consumption per hour of the electricity grid, The energy consumption of the reference system including the fossil fuel consumption by the electricity grid, can be expressed as:

25 $$F_{GSHP} = \sum\limits_{n = 1}^{8760} {F_{GSHP,grid}^{n} } .$$

where $F_{GSHP}^{{}}$ is the annual fossil energy consumption of the reference system, and $F_{GSHP,grid}^{n}$ is the amount of fossil fuel consumption per hour on the public electricity grid. Consequently, the annual energy saving rate can be written as:

26 $$P_{energy} = (FC_{GSHP} - FC_{CCHP} )/FC_{GSHP} .$$

where $P_{energy}$ is the primary energy saving rate of CCHP system, $F_{GSHP}^{{}}$ is the annual energy consumption of the reference system, and $F_{CCHP}$ is the annual fossil energy consumption of the coupled system.

(B) Reduction rate of the annual total cost

The annual total cost is chosen as the economic indicator, which includes natural gas cost, carbon tax and annual initial investment and can be calculated as follows.

27 $$CR_{CCHP} = \sum\limits_{n = 1}^{8760} {(N_{gas} (F_{pgu}^{n} + F_{b}^{n} ) + N_{grid}^{{}} E_{grid}^{n} } ) + ER_{CCHP} T{\kern 1pt} {\kern 1pt} + P\sum\limits_{k = 1}^{y} {Ca_{k} Co_{k} } .$$

where $CR_{CCHP}$ is the total annual total cost of the coupled system, $N_{gas}$ is the cost of natural gas per kilowatt per hour, $N_{grid}$ is the unit cost of the public electricity grid, $ER_{CCHP}$ is the annual CO_2_ emissions from the coupled system, $E_{grid}^{n}$ is the consumption of power in the public electricity grid, and $T$ represents the carbon tax. $Ca_{k}$ is the unit capacity of equipment in a coupled system, $Co_{k}$ is the unit cost of equipment, and $y$ is the number of pieces of equipment. $P$ is the rate of return on investment, and it could be defined in Eq. (28).

28 $$P = (I(I + 1)^{d} )/((I + 1)^{d} - 1).$$

where $I$ is the rate of interest and $d$ stands for the life of equipment; assume that $I$ and $d$ are equal for all equipment in this paper. Then the annual total cost of the reference system is shown in Eq. (29), and the reduction rate of the annual total cost can be written in Eq. (30).

29 $$CR_{GSHP} = \sum\limits_{n = 1}^{8760} {N_{GSHP} E_{GSHP,grid}^{n} } + ER_{GSHP} T + P\sum\limits_{k = 1}^{y} {Ca_{k} Co_{k} } .$$

30 $$P_{\cos t} = (CR_{GSHP} - CR_{CCHP} )/CR_{GSHP} .$$

(C) Reduction rate of the annual emissions of CO_2_

The annual CO_2_ emission rate is chosen as the environmental indicator. The CO_2_ emission rate of the coupled system includes the consumption of natural gas by the PGU and boiler, and fossil fuel consumption by the public electricity grid, which can be expressed as:

31 $$ER_{CCHP} = \sum\limits_{n = 1}^{8760} {M_{gas} (F_{pgu}^{n} + F_{b}^{n} )} + M_{grid} E^{n}_{grid} .$$

where $ER_{CCHP}$ is the annual CO_2_ emissions from the coupled system, $M_{gas}$ is the CO2 emissions per unit of natural gas, and $M_{grid}$ is the CO_2_ emissions per unit of electricity grid. The emissions of CO_2_ of the reference system include fossil fuel consumption by the public electricity grid, and can be expressed as follows.

32 $$ER_{GSHP} = \sum\limits_{n = 1}^{8760} {M_{grid} E_{GSHP,grid}^{n} } .$$

where $E_{GSHP,grid}^{n}$ is the power consumption per hour of the reference system. Therefore, the reduction rate of the annual emissions of CO_2_ can be expressed as Eq. (33) shows.

33 $$P_{environment} = {{\left( {ER_{GSHP} - ER_{CCHP} } \right)} \mathord{\left/ {\vphantom {{\left( {ER_{GSHP} - ER_{CCHP} } \right)} {ER_{GSHP} }}} \right. \kern-0pt} {ER_{GSHP} }}.$$

(D) Overall performance

To reflect the comprehensive performance of the coupled system, the energy, economic and environmental indicators are combined into the following expressions:

34 $$OB = \max \{ \delta_{1} P_{energy} + \delta_{2} P_{\cos t} + \delta_{3} P_{environment} \} .$$

where $\delta_{1}$, $\delta_{2}$ and $\delta_{3}$ are weighting factors, which require $0 \le \delta_{1}$, $\delta_{2} \le 1$, $\delta_{3} \le 1$, and $\delta_{1} + \delta_{2} + \delta_{3} \le 1$. These values represent the importance of energy, economic and environmental indicators, respectively. According to the literature, $\delta_{1}$, $\delta_{2}$ and $\delta_{3}$ are set to 1/3 equally, which means that the environmental and economic indicators are equally important. The purpose of optimizing a model is to find the maximum value of the model. In this paper, the maximization problem is transformed into a minimization problem. Therefore, the optimization objective of this model is expressed as:

35 $$P = \min (1 - (\delta_{1} P_{energy} + \delta_{2} P_{\cos t} + \delta_{3} P_{environment} )).$$

## Case study

### Building information

**Figure 4:**
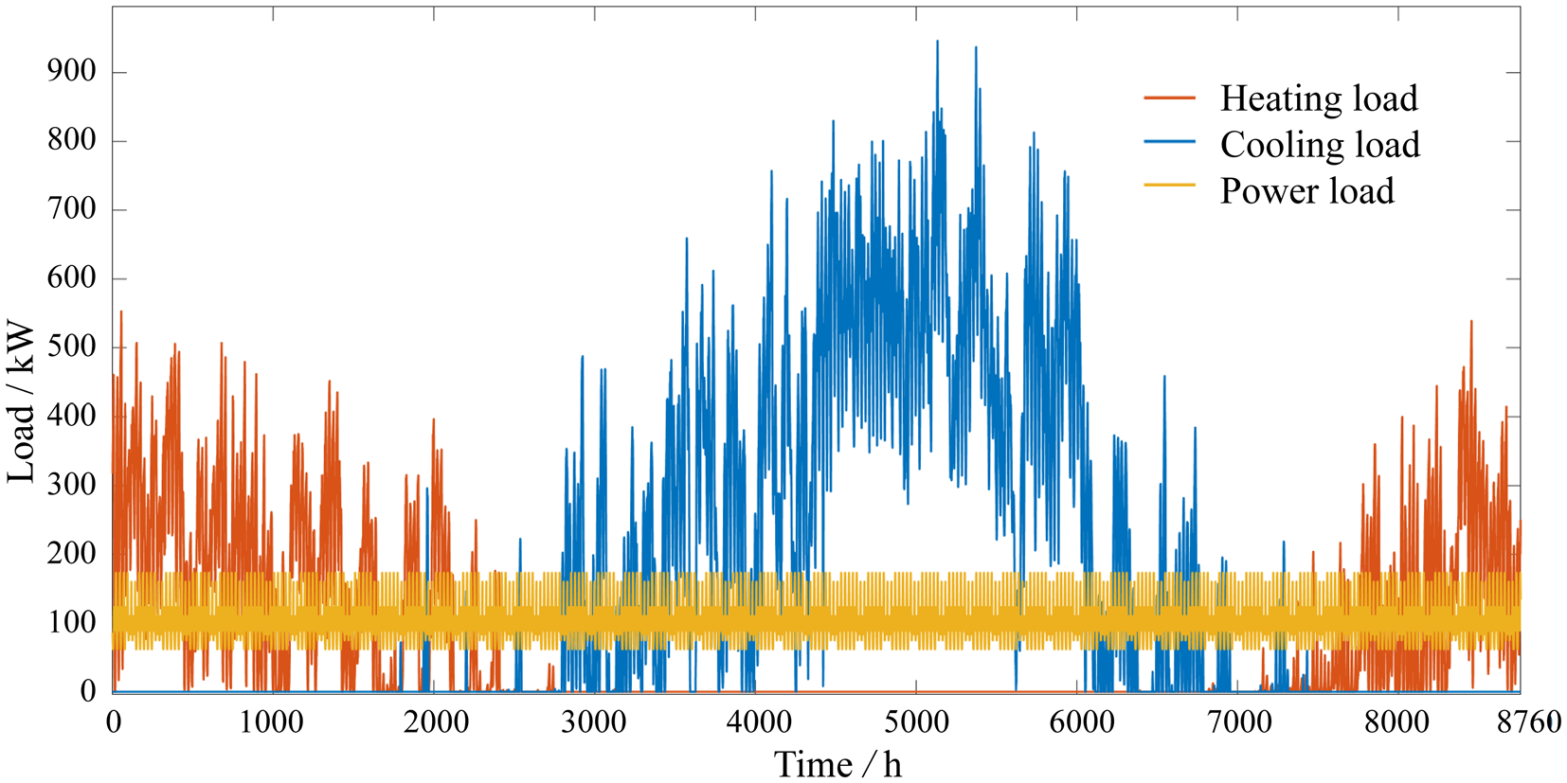
Annual loads of one building.

A hotel building is chosen to validate the proposed optimization model. The hotel building is located in Changsha city. Energy Plus software is used to simulate the hourly heating and cooling load of the building, and the results are shown in Fig. 4.

### System information

System costs and energy prices are shown in Table 1; system equipment parameters are shown in Table 2; and energy carbon emissions are shown in Table 3.

**Table 1:** System costs^23,24^.

Energy and equipment	Symbols	Price (RMB)
TOU power price (6:00–22:00)	$N_{grid}$	1.2/kwh
TOU power price (23:00–5:00)	$N_{grid}$	0.711kwh
Natural gas	$N_{gas}$	0.325/kwh
GSHP	$C_{gshp}$	2200/kw
Prime motor and regenerators	$C_{pgu + rec}$	2200/kw
Absorption refrigerator	$C_{ab}$	1200/kw
Gas-fired boiler	${\text{C}}_{b}$	300/kw
Accumulator	$C_{ba}$	2100/kw
Thermal storage tank	$C_{tst}$	230/kw
Heat exchanger	$C_{he}$	200/kw
Carbon tax	$T$	0.3/kg

**Table 2:** System equipment parameters.

Equipment parameters	Symbols	Values
Power generation efficiency of the prime motor	$E_{pgu}^{\max }$ $a_{0}$ $a_{1}$	0.39 0.049 0.7411
	$a_{2}$	− 0.4015
Efficiency of the regenerator	$\eta_{rec}$	0.8
Efficiency of the absorption refrigerator	$Q_{ab}^{\max }$	0.8
	$d_{0}$	0.425
	$d_{1}$	1.683
	$d_{2}$	− 2.419
	$d_{3}$	1.108
Efficiency of the GSHP system	$COP_{gshp}^{\max }$	4
	$a_{1}$	1.819
	$a_{2}$	− 0.819
Efficiency of the boiler	$\eta_{b}$	0.8
Efficiency of the heat exchanger	$\eta_{he}$	0.8
Efficiency of the accumulator	$\eta_{in}$	0.85
	$\eta_{out}$	1
	$\eta_{ba}$	0.2
	$\eta_{c}$	0.95
Loss coefficient of the thermal storage tank	$\eta_{tst}$	0.8
Efficiency of the electricity grid	$\eta_{E}$	0.35
	$\eta_{T}$	0.92
Efficiency of the equipment	$I$	0.08
Equipment life	$d$	20 years

**Table 3:** Energy carbon emissions parameters.

Fuel	Symbols	Values (g/kWh)
Power	$M_{grid}$	968
Natural gas	$M_{gas}$	220

## Results and discussions

### Annual power distribution of the system

**Figure 5:**
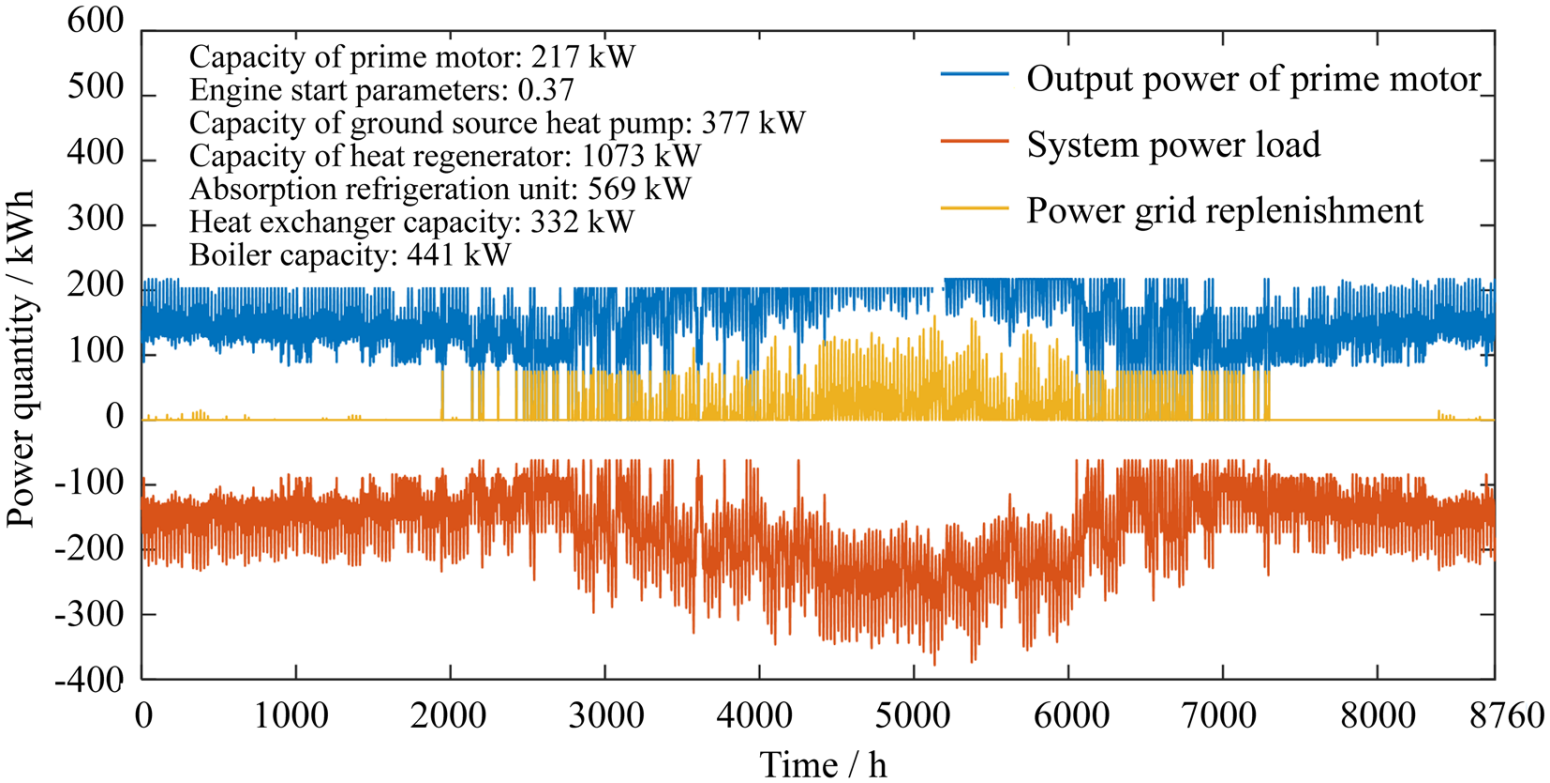
Annual power distributions of the traditional CCHP-GSHP system.

**Figure 6:**
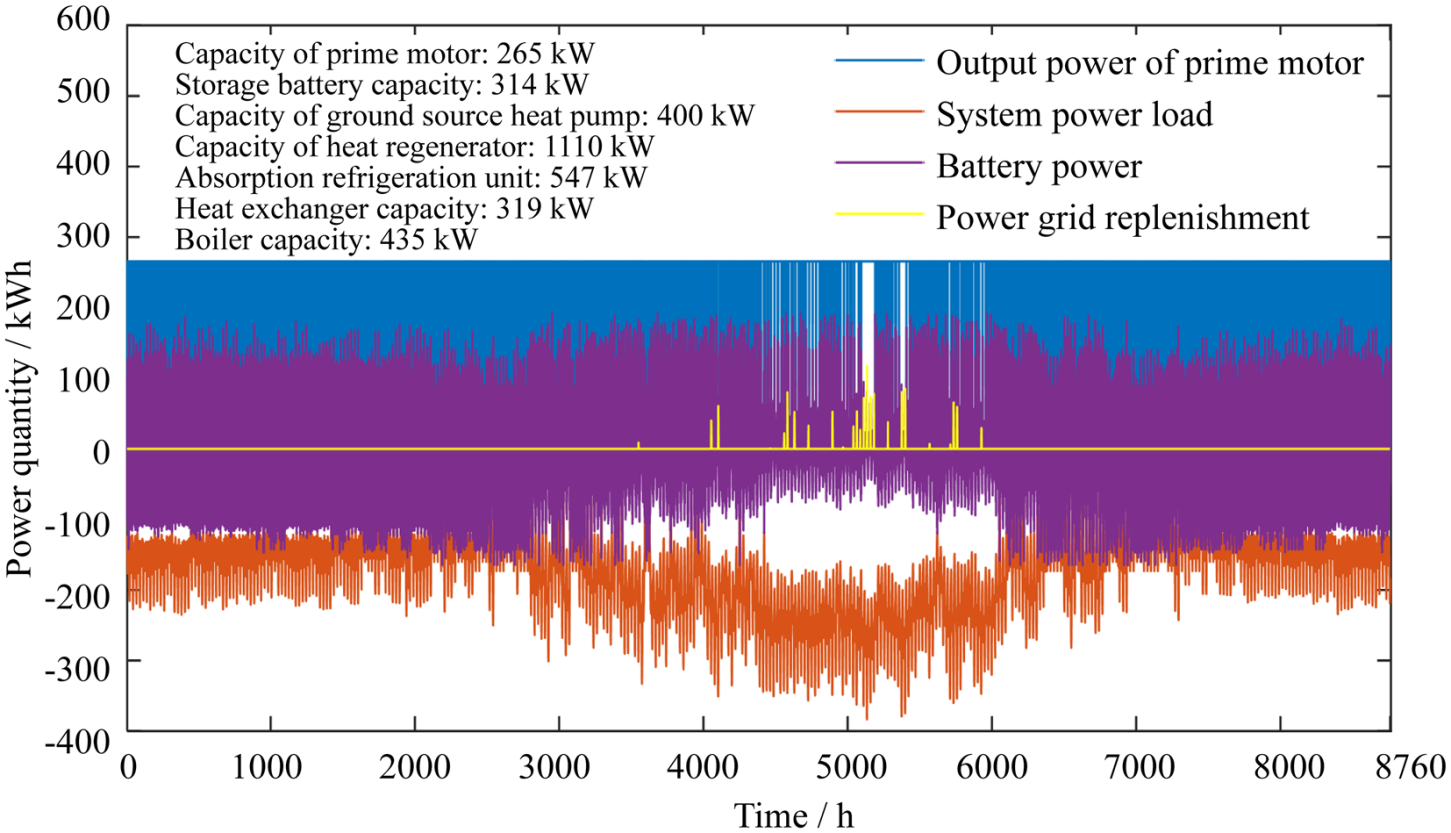
Annual power distributions of the two-point operation CCHP-GSHP system.

As shown in Figs. 5 and 6, when the PGU uses the two-point operation strategy for the CCHP-GSHP system, the power generation efficiency has the highest level and battery efficiency is relatively high, which is a good adjustment for the system. The PGU has given much support with high efficiency in the interval work. Therefore, the electricity grid replenishment space greatly decreases and the capacity of the PGU does not significantly increase. From the perspective of energy savings and environmental protection, the overall performance of the whole system is improved.

### Annual heat quantity distribution of the system

**Figure 7:**
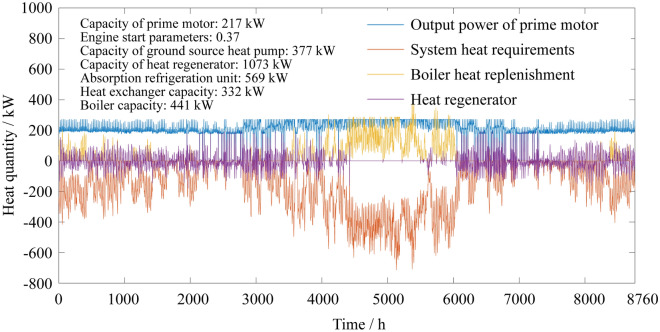
Annual heat quantity distributions of the traditional CCHP-GSHP system.

**Figure 8:**
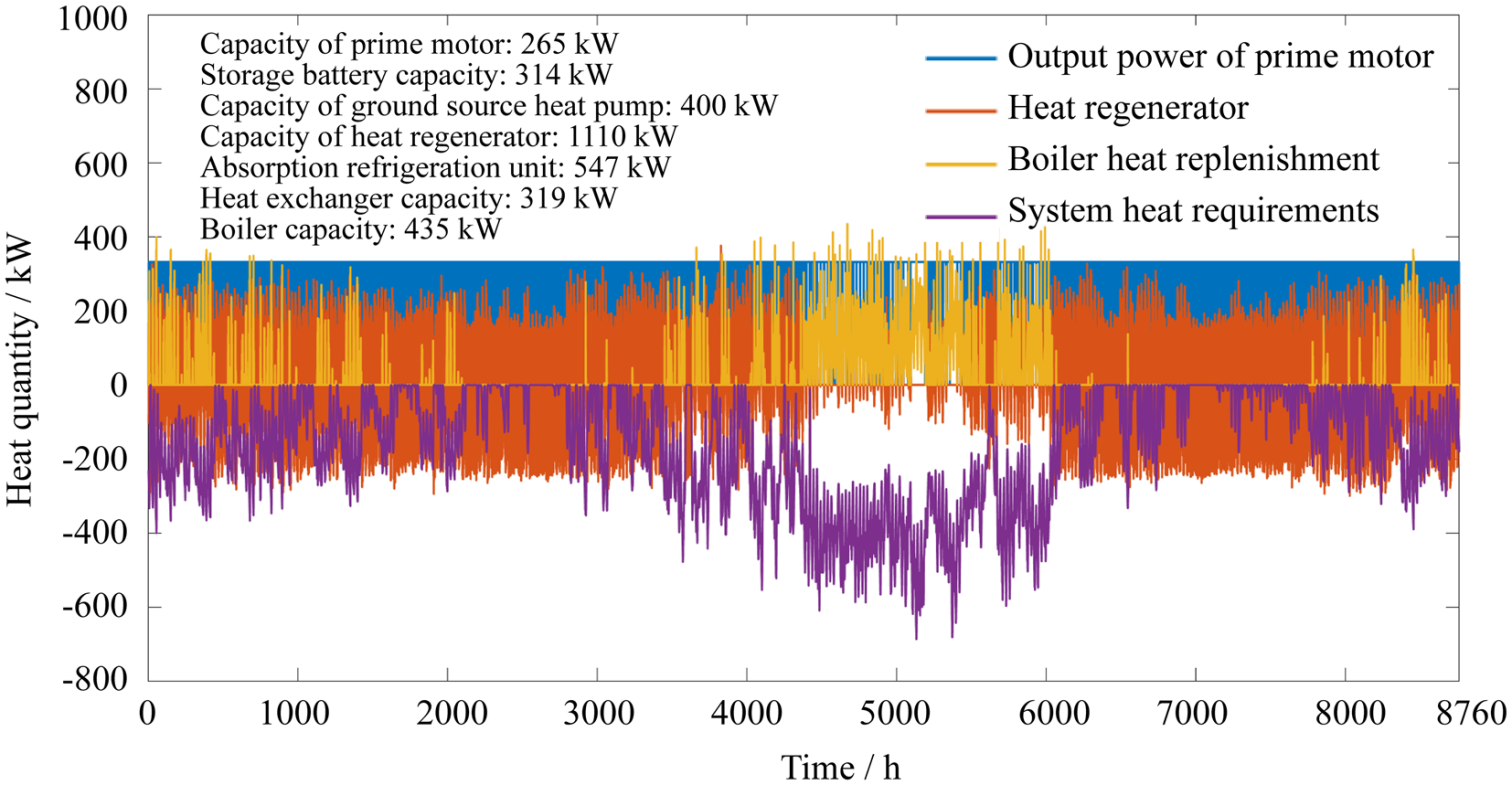
Annual heat quantity distributions of the two-point operation CCHP-GSHP system.

As seen from the comparison between Figs. 7 and 8, the PGU running at the two-point operation strategy for the CCHP system, which improves the utilization efficiency of the heat storage tank and plays a good role in regulating and providing more support for the PGU working at the high efficiency. Due to the lower economic cost of the power source in the two-point operation CCHP-GSHP system, the capacity of GSHP has been further improved. The cooling/heating cost is relatively reduced. The overall performance of the system is improved.

### Optimization indexes

**Figure 9:**
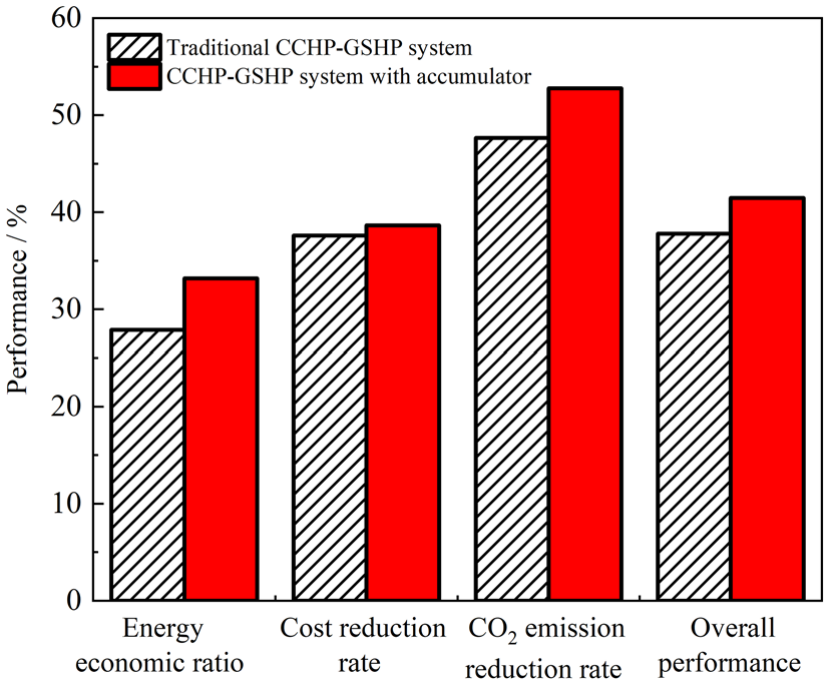
Optimization target values of two systems.

As shown in Fig. 9, compared with the reference system, the primary energy saving rate of the traditional CCHP-GSHP system is 27.7%, the annual total cost saving rate is 37.5%, the reduction rate of CO_2_ emissions is 47.7%, and the overall performance is 37.7%. The primary energy saving rate of the CCHP-GSHP system with batteries is 33.2%, the annual total cost saving rate is 38.5%, the reduction rate of CO_2_ emissions is 52.8%, and the overall performance is 41.5%. Therefore, the two-point operation CCHP-GSHP system with batteries has a 5.5%, 1%, 5.1% and 3.9% higher in primary energy saving rate, CO_2_ emission rate, annual total cost saving rate and overall performance than those of the traditional CCHP-GSHP system, respectively. Although the cost increases due to the increase in battery equipment, from the perspective of reducing fuel consumption, the performance of all aspects of the whole system has been improved.

## Conclusions

1. Compared with the traditional CCHP-GSHP system, the proposed two-point operation mode CCHP-GSHP system has advantages in all aspects, which are 5.5%, 1%, 5.1% and 3.9% higher in primary energy saving rate, carbon emission rate, annual total cost saving rate and comprehensive performance, respectively. The results show that the optimized coupling system can work in a more energy saving, environmental protection and economic way and prove the effectiveness of the coupling system, operation strategy and optimization method.
2. To satisfy the building load fluctuation and strengthen the combination performance of the coupled system, this paper provides a certain reference and suggestion for subsequent CCHP-GSHP system integration and operation strategy.

## Data Availability

The datasets analyzed during the current study are available from the corresponding author on reasonable request.
